# Deep Super-SAGE transcriptomic analysis of cold acclimation in lentil (*Lens culinaris* Medik.)

**DOI:** 10.1186/s12870-017-1057-8

**Published:** 2017-06-30

**Authors:** Abel Barrios, Constantino Caminero, Pedro García, Nicolas Krezdorn, Klaus Hoffmeier, Peter Winter, Marcelino Pérez de la Vega

**Affiliations:** 1 Instituto Tecnológico Agrario de Castilla y León, Consejería de Agricultura y Ganadería, Junta de Castilla y León, Finca Zamadueñas, Ctra. Burgos km, 119, 47071 Valladolid, Spain; 2 Area de Genética, Departamento de Biología Molecular, Universidad de León, 24071 León, Spain; 3 GenXPro, Altenhöferallee 3, D-60438 Frankfurt am Main, Germany; 4 Present Address: Escuela Universitaria de Ingeniería Agrícola I.N.E.A, Con. Viejo de Simancas, Km. 4.5, 47008 Valladolid, Spain

**Keywords:** Lentil, Deep Super-SAGE, Gene expression, Frost, Stress, Cold acclimation

## Abstract

**Background:**

Frost is one of the main abiotic stresses limiting plant distribution and crop production. To cope with the stress, plants evolved adaptations known as cold acclimation or chilling tolerance to maximize frost tolerance. Cold acclimation is a progressive acquisition of freezing tolerance by plants subjected to low non-freezing temperatures which subsequently allows them to survive exposure to frost. Lentil is a cool season grain legume that is challenged by winter frost in some areas of its cultivation.

**Results:**

To better understand the genetic base of frost tolerance differential gene expression in response to cold acclimation was investigated. Recombinant inbred lines (RILs) from the cross Precoz x WA8649041 were first classified as cold tolerant or cold susceptible according to their response to temperatures between −3 to −15 °C. Then, RILs from both extremes of the response curve were cold acclimated and the leaf transcriptomes of two bulks each of eight frost tolerant and seven cold susceptible RILs were investigated by Deep Super-SAGE transcriptome profiling. Thus, four RNA bulks were analysed: the acclimated susceptible, the acclimated tolerant and the respective controls (non-acclimated susceptible and non-acclimated tolerant). Approximately 16.5 million 26 nucleotide long Super-SAGE tags were sequenced in the four sets (between ~3 and 5.4 millions). In total, 133,077 different unitags, each representing a particular transcript isoform, were identified in these four sets. Tags which showed a significantly different abundance in any of the bulks (fold change ≥4.0 and a significant *p*-value <0.001) were selected and used to identify the corresponding lentil gene sequence. Three hundred of such lentil sequences were identified. Most of their known homologs coded for glycine-rich, cold and drought-regulated proteins, dormancy-associated proteins, proline-rich proteins (PRPs) and other membrane proteins. These were generally but not exclusively over-expressed in the acclimated tolerant lines.

**Conclusions:**

This set of candidate genes implicated in the response to frost in lentil represents an useful base for deeper and more detailed investigations into this important agronomic trait in future.

**Electronic supplementary material:**

The online version of this article (doi:10.1186/s12870-017-1057-8) contains supplementary material, which is available to authorized users.

## Background

Lentil (*Lens culinaris* Medik. subsp. *culinaris*) is a founder crop of modern agriculture and one of the earliest domesticated plant species in the Fertile Crescent. It is a diploid (2n = 14), self-pollinating annual cool season grain legume normally grown in temperate semi-arid regions, usually in rotation with cereals. It plays an important role in human nutrition and soil improvement contributing to replenishing soil nitrogen levels. The crop is now cultivated throughout Western Asia, Northern Africa, the Indian subcontinent, Australia and North America [1, 2]. This wide area of cultivation includes regions with frosty winters, such as Central Turkey, Central Spain and the North American Pacific Northwest. A strategy to improve lentil yield in these areas is early or winter sowing. Increases in yield due to winter sowing ranged from 20% to 100% in field trials in different countries and climatic conditions. Early sowing requires well-adapted winter-hardy, freezing tolerant cultivars [3]. Thus, increasing frost and cold tolerance are major objectives of international lentil breeding programs. The inheritance of radiation frost tolerance has been suggested to be monogenic in lentil [4]. Field tests revealed that lentils that do not undergo cold acclimation prior to frost are unable to survive freezing temperatures later on [5].

Low temperature is a key environmental factor limiting plant growth and distribution. To cope with cold stress, plant species have evolved physiological and molecular adaptations to maximize cold tolerance including gene expression regulation [6]. Chloroplast is the first and most severely affected organelle when plants are exposed to frost and it is the target of many cold acclimation processes [7]. As consequence of these mechanisms plants are able to survive exposure to low temperature via a process known as cold acclimation or chilling tolerance. Cold acclimation is a progressive acquisition of freezing tolerance (FT) by plants subjected to low, non-freezing temperatures. Cold acclimation serves two major functions: the adjustment of metabolism and basic cellular function to the biophysical constraints imposed by such temperatures (so called ‘chilling responses’), and the induction of FT (‘FT responses’). The mechanisms involved in cold acclimation are related to a number of processes that include: modifications of plant cell membranes, changes in photosynthesis levels and photosynthesis-related components, and differential expression of several gene sets such as cold-responsive genes (COR) and a family of transcriptional activators called C-repeat binding factors (CBFs), also known low temperature responsive elements (LTREs) [6–9].

Kahraman et al. [10, 11] conducted a field study on winter hardiness in several environments using several lentil Recombinant Inbred Lines (RILs) including the same RIL used in the present study. Parental line WA8649041 was the most winter hardy. Heritability estimates of winter hardiness among RIL populations ranged from 15.9 to 90.7%. They further mapped quantitative trait loci (QTL) for winter hardiness in a population obtained with one of the parents used in the current work (Precoz) and demonstrated that tolerance to low temperature is a multi-genic trait. Seven QTLs were identified for winter hardiness, with only one common QTL across all environments [11].

As a follow-up to this initial work, the aim of the present study is to identify genes differentially expressed in lentil in response to cold acclimation or chilling tolerance using genome-wide transcriptome profiling. The plant material we selected for this study are lentil RILs which have been used in previous studies on frost response and other studies [12]. For transcript quantification we here employ Deep Super-SAGE [13–15], a tag-based transcript quantification method that utilizes the same principles as the Serial Analysis of Gene Expression method (SAGE) [16] but provides major improvements. Especially it generates 26 bp long so called Supertags from each transcript that can be much more reliably annotated to known gene sequences than the 14 bp long SAGE tags. Moreover, since Deep Super-SAGE tags are sequenced by new-generation-sequencing technology, transcription profiles are more comprehensive than the Sanger-sequencing based SAGE profiles. Deep Super-SAGE was selected because it was a reliable and available technique when this experiment was designed and implemented in 2008. Although Deep Super-SAGE yields a lower number of data than the now current choice technique, RNA-seq, it is able to detect low-copy transcripts and the number of millions of tag is high enough to detect expression changes related to the candidate genes as it has been demonstrated in other legume species [14, 15].

We here apply this technology for the analysis of gene expression of a set of candidate genes that are differentially expressed in leaves of cold-tolerant and susceptible lines of lentil and in tolerant and susceptible bulks from RILs.

Recent advances in genomics, transcriptomics and other ‘omics’ offer unprecedented opportunities to address the question of what genes are involved in the plant’s response to biotic or abiotic stresses. New generation sequencing techniques and microarrays have contributed to widening the use of these ‘omics’. The result of these techniques is the identification of candidate genes involved in these responses [17, 18].

## Methods

### Materials

The lentil (*Lens culinaris* Medik. subsp. *culinaris*) genotypes used in this study were 90 F6-7 RILs from the cross Precoz (frost susceptible) x WA8649041 (tolerant). The tolerant Syrian landrace ILL4400 [19] was included as tolerant check. WA8649041 (PI 547039) is a known source of winter hardiness. It is a breeding line obtained in the USDA-ARS breeding program at the Washington State University, Pullman [20], has a prostrate plant structure, very strong branch habit, microsperma seed with orange cotyledons, brown seed coat color and late flowering. Precoz (ILL4605) is a cold-susceptible macrosperma cultivar originated in northern Argentina [21]. It is moderately branched and semi-tall erect with yellow cotyledon, green seed coat colour and early flowering [22]. Precoz, WA8649041 and the corresponding RILs were kindly provided by Prof. F. Muehlbauer (Washington State University, Pullman, Washington, USA). WA8649041 is freely available as germplasm without restrictions from the USDA-ARS Plant Germplasm System under the accession number PI 547039. Precoz was obtained from Center for Agricultural Research in the Dry Areas (ICARDA) formerly in Aleppo, Syria; it is registered and available from ICARDA as accession ILL4605. The ILL4400 accession was also provided by ICARDA.

### Response to cold temperatures

**Fig. 1:**
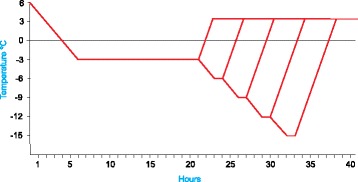
Scheme of the cold treatments to determine the level of frost response of the Precoz x WA8649041 RILs

First, RILs were scored in relation to the response to frost in a cold chamber. Eighteen seeds per RIL and frost treatment were sown in pots containing a 2:1:1 mixture of peat, sand and vermiculite. The frost treatments were repeated twice and two pots per RIL and treatment were used, thus a total of 36 plantlets per RIL and frost level were scored. After a three-week cold-acclimation period at 6 °C/12 h light and 4 °C/12 h dark cycle, groups of plantlets were subjected to −3, −6, −9, −12 and −15 °C in the dark. The temperature was lowered 1.5 °C per hour to reach −3 °C and plantlets were kept 15 h at this temperature, then, for more intense frost, the temperatures were lowered at the same rate and plantlets kept 1 h at each level of temperature as shown in Fig. 1. After the final frost treatment the temperature for each set of plantlets was increased to 4 °C at a rate of 4 °C per hour. Plantlets were kept at this temperature for 24 h in the dark (Fig. 1), after that, temperature and light conditions increased in the four following days to reach 6 °C in light (0.5 °C/day) and 4 °C in dark with a 12 h light/dark cycle (3 h/day). The final read-out was the survival of the plantlets after each cold treatment after 4 weeks of recovery. Additional files 1 and 2 show some examples of the response.

**Fig. 2:**
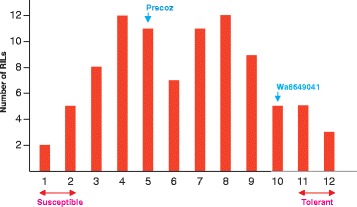
Number of Precoz x WA8649041 RILs in each of the 12 levels of response to frost. Arrows indicate the level shown by the two parents. Double-headed red arrows indicate the pairs of levels considered as susceptible or tolerant for the Deep Super-SAGE experiment. Two set of bulked RNA were obtained, one from the eight tolerant RILs and another one from the seven susceptible RILs

After the 4 weeks of recovery, the survival rate of each set of 18 plants was evaluated (two values per RIL and treatment). An analysis of variance showed that there were significant differences (*P* < 0.01) among RILs at all temperatures except at −3 °C; the data at this temperature were not included in further analyses. Groups of RILs with similar response were estimated by the hierarchical Ward’s cluster analysis [23] (Ward’s minimum variance criterion minimizes the total within-cluster variance). According to the Ward’s analysis result, the Tukey multiple comparison of means method (considering *P* < 0.05) was used to evaluate differences between groups [24]. The results of the tests defined 12 groups of RILs (Fig. 2) so that there were no significant survival differences within each group but there were significant differences between groups, at least at one of the four temperatures. The two groups at each extreme of the distribution were considered as susceptible and tolerant respectively (Fig. 2).

### RNA isolation and construction of the Deep Super-SAGE libraries

According to the frost tolerance tests seven RILs were considered as susceptible and eight as tolerant. These RILs formed the bulks for Deep Super-SAGE transcriptome profiling later on. Eighteen seeds per RIL were sown as previously described. Seedlings were grown at 20 °C in light and 16 °C in dark, in a 12 h each dark/light cycle until they reached the two-leaf stage. Then, half of the seedlings were transferred to cold acclimation at a 6 °C/12 h light 4 °C/12 h dark cycle for 3 weeks, whereas the other half was kept at the initial conditions. After this period, the green plantlets were frozen in liquid nitrogen and then stored at -80 °C overnight prior to extraction of total RNA from the leaves. Plant material was homogenized and RNA from each RIL and treatment was extracted by the TRIZOL® procedure according to manufacturers (ThermoFisher) recommendations. RNA was quantified by using a Qubit spectrophotometer and an equal quantity from each RIL was bulked. Thus, four bulks of RNA were obtained: acclimated susceptible (AS), acclimated tolerant (AT), non-acclimated susceptible (NAS) and non-acclimated tolerant (NAT). Subsequent steps for the construction of Deep Super-SAGE libraries were performed as detailed by Matsumura et al. (2003) [13]. However, instead of concatenation of di-tags and subsequent cloning and sequencing, amplified ditags were directly sequenced by 454 Life Sciences, Branford, CT, USA [14].

### Supertag quantification and data analysis

For each library, 26 bp long Supertags were extracted from the sequences using the GXP-Tag sorter software. Library comparison and primary statistical treatment was carried out using the DiscoverySpace 4.01 software (Canada’s Michael Smith Genome Sciences Centre, available at http://www.bcgsc.ca/discoveryspace). Scatter plots of the distribution of the expression ratios (R_(ln)_) and significance levels of the results were calculated according to Audic and Claverie [25].

### Sequence homology alignment

Supertag sequences were used to search for lentil sequences in the NCBI database using the program BLAST [26] (E value cut of ~3 e^−7^) and EST sequences obtained from several lentil accessions by the University of Leon and GenXPro. Lentil sequences identified with a similarity ≥92% by the Supertags were used in BLASTN search against genomic and EST sequences from the closely related (E value cut of ~ e-^−8^), well researched legumes, *Medicago truncatula, Cicer arietinum* and *Glycine max* for sequence alignment and assignment of potential function. Information from other legumes species was used when appropriate. In addition, the deduced amino acid sequences of the identified lentil sequences were used in a BLASTP search against the UniProt database for assignment of potential function.

## Results

The distribution of the RILs in relation to the 12 levels of response to frost showed a transgressive segregation (Fig. 2). At −3 °C almost all RILs survived and frost damages as e.g. leaf and/or meristem necrosis were almost negligible; thus, this temperature was not considered for further analyses. At lower temperatures the plants showed differential survival rates and damage levels which allowed defining frost susceptible and frost tolerant lines.

**Fig. 3:**
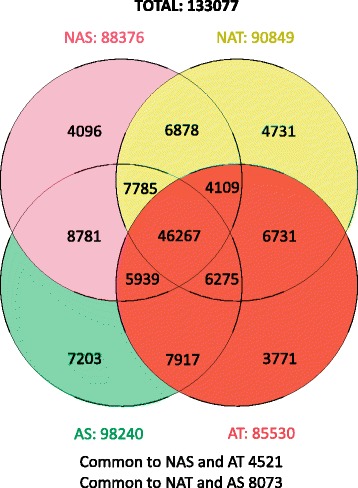
Number of unitags found in each of the lentil RNA bulk sets or common to two or more sets

Approximately 16.5 million 26 nt long Supertags were sequenced in the four sets. A total of 4,220,553 tags were sequenced from the non-acclimated susceptible RIL bulk (NAS), 4,011,993 from the non-acclimated tolerant RIL bulk (NAT), 5,357,611 from the acclimated susceptible bulk (AS) and 2,990,512 from the acclimated tolerant bulk (AT). In total 133,077 different unitags, i.e. tags that represented a group of identical tag sequences representing a particular mRNA isoform, were identified in these four sets. Figure 3 shows the distribution of these unitags among the four sets; those observed only in a single set and those common to two or more sets. Since the total number of tags sequenced in each of the four libraries was different, the observed numbers of unitags were normalized in each library to tags per million [(quantity of a unitag/total number of tag in the library) * 10^6^]. Absolute numbers of individual tags ranged from one to 8318 and the normalized numbers from 0.19 to 1,552.56 per million. The normalized number of each unitag in each combination (genotype-treatment) represents an estimation of the gene expression level in that situation. Comparisons between combinations were carried out with normalized numbers. The mean and standard deviation of the four normalized values of each tag were calculated.

To find putative sequences differentially expressed between sets, tags very unevenly distributed between the four sets of data were searched. First, tags with a coefficient of variation (ratio between the standard deviation and the mean) higher than 1.5 were selected. To eliminate underrepresented tags only those with an absolute number equal to or higher than 50 were considered. A total of 450 unitags fitted these conditions. Among these tags, the first search was directed to identify unitags only observed in one of the four combinations (NAS, NAT, AS or AT) with a fold change ≥4.0 and a significant *p*-value (*P* < 0.001). The numbers of tags exclusively expressed in one of the four conditions ranged from 43 (AT) to 80 (AS) (Table 1) with a total of 257. A second search was carried out for those tags which showed a fold change higher than 4.0 between resistant vs. susceptible lines irrespective of the treatment, or between treatments (acclimation vs. non-acclimation) irrespective of the tolerance or susceptibility of the lines. In this second search some tags were common in two combinations.

**Table 1:** Summary of the number of significant tags and the lentil identified gene sequences

	Number of significant tags	Number of tags identifying lentil sequences
Only observed in:		
Acclimated susceptible (AS)	80	44
Acclimated tolerant (AT)	43	30
Non-acclimated susceptible (NAS)	67	44
Non-acclimated tolerant (NAT)	67	49
Over-represented in:		
Non-acclimated (N)	36	29
Acclimated (A)	58	35
Tolerant (T)	52	29
Susceptible (S)	61	40

Tags that fitted these criteria are listed in Additional files 3, 4, 5, 6, 7, 8, 9 and 10 together with their absolute and normalized numbers and the fold change value in each comparison. The genes represented by these tags were identified by BLAST, first against lentil sequence databases and then to other legume sequences. Additional files 11, 12, 13, 14, 15, 16, 17 and 18 summarize the lentil and legume genes identified and, when known, their most likely function.

Identified lentil sequences from the four bulks (NAS, NAT, AS or AT) were functionally annotated by using the Blast2GO software. Some of the sequences were not long enough for a full annotation and were included in the not-assigned group. Among the annotated sequences, the most abundant in all the four set of data were sequences related to photosynthesis and protein synthesis and metabolism. Unfortunately, the number of sequences in other categories was too low to carry out meaningful comparisons.

There were 300 unitags (65%) (Table 1 and Additional files 1, 2, 3, 4, 5, 6, 7 and 8) that identified gene sequences in lentil (or in other close related legume species such as pea). Two criteria were used to consider a positive identification: 1) at least 22 out of the 26 nt of the tags had to match the lentil sequence, and 2) the four nucleotides of the endonuclease target at the 5′ end of the tag had to be present in the lentil sequence. In the large majority of the positive matches the homology was between 24 and 26 nt. Mismatches are probably due to the fact that sequences in databases come from different lentil accessions. Another source of mismatches is due to the 3′ UTR origin of many tags. A high proportion of Super-SAGE tags are obtained from the 3′ end of cDNAs, which means that many tags come from the 3′ UTR, where polymorphism is higher than in the coding sequence.

Sometimes the same lentil gene sequence was identified by two or more different tags. This is generally due to the presence of two or more endonuclease targets along the gene sequence, generating different 26 nt tag sequences along the lentil gene sequence. This was particularly notable in transcripts from the chloroplast genome.

The BLAST results also displayed different tags that match with different lentil sequence sets but corresponding to the same protein function. For example, tags AS11 and AT26 identified different lentil sequences with similarity to different *Medicago* phospholipase Ds (Q2HUA3_MEDTR and G7JYS0_MEDTR); or AT21 and NAT29 were related to non-specific serine/threonine protein kinases.

At times tags identified members of heterogeneous sequence families increasing the difficulty to identify particular genes from tag sequences. This is the case of glycine-rich or putative glycine-rich protein genes, also designated as cold and drought-regulated protein CORA or dormancy-associated protein, and that share high sequence similarity among them. A set of tags (NAT7, NAT46, A1 = T1, A7 = T4, A8 = T28, A12, A18 = T25, A20, A22, A25 = T26, A32, and A35) identified lentil sequences which share similarity with different genes, all of them included in this heterogeneous glycine-rich family (Table 2). These tags were predominantly observed in the acclimated tolerant lentils and some in the non-acclimated tolerant. Thus they seem to be collectively overexpressed in the tolerant genotypes.

**Table 2:** Tags related to glycine-rich and cold and drought sequences and their distribution

	NAS	NAT	AS	AT
CATGATGCTCATTCAAAGTAATAAGT NAT7		**94.47** ^**a**^		
CATGATGCAAATTCAAAGTAATAAGT NAT46		**14.21**		
CATGAATCTTTTGAGGTTATGTATTA A1 = T1		0.25		**2268.51**
CATGAATCTTGTATGGTTATGTATTA A7 = T4		1.25		**158.50**
CATGTATATGTAGTAACTAGTTTAAT A8 = T28	0.47	0.25	3.17	**61.86**
CATGCACGTATTAAGTGTAATATAAA A12	8.77	98.21	668.21	**6150.45**
CATGGTTATAGCCTCCTCCTCCACCA A18 = T25, Antisense			0.93	**20.06**
CATGTGTAATTTATCAAATATCATCA A20	19.43	68.54	537.55	**2591.53**
CATGGTTTTTCTCATTTCATCAGTGG A22	0.24	0.25	1.31	**15.38**
CATGATGATATAATCAGTTACCATTG A25 = T26, Antisense			0.75	**15.38**
CATGAATAAATAGAGTTGGATGTTGA A32	0.24	2.24	6.16	**43.80**
CATGGCCAATAGGCCAAGGATGAGGA A35 Antisense	0.24	1.25	2.99	**21.40**
Total	29.39	280.92	1221.07	**11,346.87**
Total antisense	0.24	1.25	4.67	**56.84**

^a^Numbers in bold indicate the highest value for each tag, normalized values. Empty cells indicate zero observations

The majority of the tags were translatable to the corresponding protein as classified as sense transcripts, however, nine tags were identified as antisense transcripts (Table 3). Tag NAS10/10a is located in the spacer between two close genes which are transcribed in opposite directions, the antisense NAS10a corresponds to a haloacid dehalogenase-like hydrolase. While in the sense direction would be part of a peptidyl-prolyl cis-trans isomerase transcript. In barrel medic the two homolog sequences are closely located (1g085560 and 1g085570, spaced 451 pb) and are transcribed from different DNA chains (Mt4.0v1). Three tags (A18 = T25, A25 = T26, A35) were related to sequences of the heterogeneous group of the CORA glycine-rich proteins and were almost exclusively observed in the acclimated plants and mainly in the tolerant RILs. On the contrary, the antisense tags related to the protease inhibitor/seed storage/LTP family protein and the Protein FAM135B-like were exclusively or almost exclusively observed in the non-acclimated plants of the tolerant RILs (NAT38, N15 = T30) (Table 3).

**Table 3:** Tags identifying antisense sequences

TAG number	TAG sequence	Fold change^1^	Nucleotide sequence identity between TAG and lentil sequences^2^	*Lens* sequences	Other legume species sequences	UniProt identification and accession number
AS8	CATGAGATTGCACTTGAGTAACTTGG ANTISENSE	7.34	25-26	64544 647 331115122	502080648 2 g104400	Mediator of RNA polymerase II transcription subunit 15a?? Transcription cofactor, putative, G7IM96_MEDTR??
AS27	CATGAGTTGGCGTCCAATATTTTGAA ANTISENSE	5.67	25-26	72001 296 56138 122 331117328	086s0008 502127601	ABC transporter C family member??
NAS10a	CATGTTTTTGAATTGTGATTGCTCTC ANTISENSE	7.28	25	N107087 N6881 331108837 368545933	388509589 502117510 571480914	Haloacid dehalogenase-like hydrolase, A0A072VYK9_MEDTR
NAT38	CATGATTTTCCCTCTCAAGACTAAGT ANTISENSE	5.10	25-26	N11063 68382 215 66867 237	552990588 502124263 571537824	Protein FAM135B-like protein, A0A0B2PEB3_GLYSO
NAT39	CATGGTCATAAAGGTTCTGAATAGGG ANTISENSE See AS2 and AT7	5.10	25-26	331108296 268537762 268541446	5g097280 502099852 359806052	Light-harvesting complex I chlorophyll A/B-binding protein, G7KEN2_MEDTR Chlorophyll a/b binding protein 215, CB215_PEA
N15 = T30	CATGATGTCGCCGACCGTAACAATAA ANTISENSE	5.08	25-26	331109463 87696 239 64868 288	1g012710 1g012690 502124985	Protease inhibitor/seed storage/LTP family protein, G7I725_MEDTR
A18 = T25	CATGGTTATAGCCTCCTCCTCCACCA ANTISENSE See A1, A22 and A35	5.39	26	331121144 65367 194	5g084570 114415397 502097761	Cold and drought-regulated protein CORA, G7K7D2_MEDTR Putative glycine-rich protein, Q0E7L3_PEA
A25 = T26	CATGATGATATAATCAGTTACCATTG ANTISENSE	5.01	23-26	65697 176 331109145	2605888	Dormancy-associated protein, O22612_PEA (glycine-rich protein).
A35	CATGGCCAATAGGCCAAGGATGAGGA ANTISENSE See A1, A18 and A22	4.04	26	C1949 331121144 331108748	5g084570 114415397	Putative glycine-rich protein, Q0E7L3_PEA Cold and drought-regulated protein CORA, CORA_MEDSA G7K7D2_MEDTR

^1^ The 0 normalized values were changed to 0.5 to calculate fold change

^2^ Two numbers indicate the existence of polymorphism among lentil sequences in the nucleotide sequence corresponding to the TAG

These antisense transcripts could be a source of natural derived siRNAs or natural antisense short interfering RNA natsiRNAs, thus the expression of antisense transcripts could result in the downregulation of the corresponding genes.

The set of tags exclusively observed in acclimated susceptible RILs **(**AS) included a total of 80 tags among which 44 identified lentil sequences (Additional files 3 and 11). This group includes nuclear sequences related to photosynthesis and other chloroplast functions (e.g., psbY, chlorophyll a-b binding protein, Additional file 11). This result could suggest a differential transcriptional and translational activity related to chloroplast functioning. A set of tags are related to abiotic stress response, including response to cold (aquaporin, phospholipase D, germin-like protein, ABC transporters, cold-acclimation specific protein 15, and late embryogenesis abundant protein-LEA). At least some of these proteins, such as aquaporins, ABC transporters, and LEA, are membrane components and are involved in stress response. Finally, a tag identified a glycine degradation enzyme, glycine dehydrogenase P protein; glycine enzymes implicated in the degradation pathway have been described as constitutive differences between pea genotypes in relation to cold response [27].

The acclimated tolerant exclusive tag set (AT) included a total of 43 overrepresented tags, and 30 of them identified lentil sequences (Additional files 4 and 12). Approximately 27% of the tags are related to chloroplast functions or mitochondrial functions (Additional file 19). Two of most overrepresented tags (AT1 and AT2) were related to plant hormone-induced proteins: an auxin induced proline-rich protein and an abscisic acid induced LEA domain protein. Two other tags identified components of membranes. Finally four tags identified gene sequences that are likely implicated in cold and other abiotic stress responses (acyl carrier protein, LEA, phospholipase D, aluminum induced protein). When the tags overrepresented in the AT bulk were added to the tags exclusively observed in this AT bulk, the number of sequences related to abiotic stress increased due to the incorporation of several glycine-rich CORA proteins (see end of Additional file 12, tags added from other tables).

The non-acclimated exclusive susceptible tag set (NAS) included a total of 67 tags, and 44 identified lentil sequences (Additional files 5 and 13). Similar to the AT tag set the NAS tag set included sequences (~ 14%) related to photosynthesis and chloroplast metabolism (Additional file 19). Three tags (NAS6, NAS7 and NAS40) identified stress-related sequences regulated by gibberellins, identified as snakins. Although the three lentil sequences matched with a single database sequence from other legume species, a detailed sequence analysis after alignment indicated that there are at least two different gene sequences in lentil (NAS 7 and NAS40 identified the same lentil sequences). This result agrees with *M. truncatula* where two genes are described (1g025250 and 1g025220). Other groups of sequences were related to response to stresses (heat shock protein, GDSL-like lipase/acylhydrolase, HVA22-like protein a, beta-glucosidase, ascorbate peroxidase). Several tags related to photosystems and chloroplast functioning were added to this group from the N and S groups of tags (Additional file 13).

The non-acclimated exclusive tolerant tag set (NAT) consisted of 67 tags, of which 49 matched lentil database sequences (Additional files 6 and 14). The over expressed lentil sequences related to photosynthesis and other chloroplast functions were less frequent (~ 8%) in this tag set. They were limited to chlorophyll a/b binding protein (NAT25, NAT39 and NAT45) and phytol kinase, A0A072UY92_MEDTR (NAT44) an enzyme involved in the activation and reutilization of phytol from chlorophyll degradation. Conversely, some of them coincide with sequences identified by tags expressed under the other conditions. NAT39 coincides with a sequence identified in both genotypes under acclimation conditions, AS2 and AT7, although NAT39 matched the antisense sequence.

A relatively large number of NAT tags (13, ~ 27%) represented diverse, known stress-responsive genes (Additional file 19), including pathogenesis-related (PR) genes. Tag NAT49 is likely related to vernalization response (vernalization insensitive-like protein, A0A072UMI5_MEDTR). Tag NAT22 matched with a conserved nucleotide sequence within two similar thaumatin-like gene sequences which are clearly different members of a lentil gene family; the two lentil sequences best fit a single sequence in *Cicer* and in *Medicago* in the UniProt database, which are included in the PR proteins (PR-5a in *Cicer*, O81927_CICAR; A0A072TVX9_MEDTR). The sequence identified by NAT16 was homologous to another member of the PR gene family, an allergen protein gene (H9BPI0_VICFA) from *Vicia*. Both sets of lentil sequences seem to be polymorphic, but while those related to NAT16 seems to be allelic on the basis of their sequence similarity, those related to NAT22 could be subdivided in two groups of sequences. If these two groups are allelic or members of a gene family would need further genetic analysis.

Six of the identified functions are related to protein synthesis, folding and metabolism, in addition to ribosomal proteins (Additional file 19). One of these functions, Cu-Zn-superoxide dismutase copper chaperone (NAT43), could contribute to increase the final activity of a stress-related protein, Cu-Zn superoxide dismutase (NAT32), included among stress-responsive proteins.

A further comparison was the analysis of the overrepresented tags considering two different sets of data: 1) acclimated vs. non-acclimated lentil plants, irrespective of their response to frost; 2) susceptible vs. tolerant RILs, irrespective if they were acclimated or not. The first set would represent those functions generally over-expressed in response to acclimation in lentil, while the second would represent genotype-dependent gene-expression independently of cold acclimation.

Among the 36 tags overrepresented in the non-acclimated lentil plants (named as N), susceptible or tolerant, 29 identified lentil sequences (Additional files 7 and 15). Most of the tags (15) matched to sequences of the chloroplast genome, either ORF sequences or gene spacer sequences; one identified a mitochondrial sequence, another a protein of organelle ribosomes, and the rest identified nuclear gene sequences including one coding for the small subunit of the ribulose bisphosphate carboxylase. Most of the chloroplast sequences were directly related to photosystems, cytochromes and the ribulose bisphosphate carboxylase large subunit.

Among the 58 tags overrepresented in the acclimated lentil plants (named as A), susceptible or tolerant, 35 identified lentil sequences (Additional files 8 and 16). A majority of the identified sequences (~ 23%) were included in two diverse groups of glycine-rich and proline-rich proteins (Tables 2 and 4). The definitive identification of particular gene was especially difficult in the glycine-rich protein group, since the repetitive nature of these sequences. Within this group are included sequences related to proteins which have been functionally described as cold- and drought-regulated proteins (CORA) or as dormancy associated proteins. Similarly, the desiccation protectant protein LEA14-like (A24) and the low-temperature inducible protein (A30) could be related to the adaptation to cold. Two tags identified pathogenesis-related genes, the lentil PR4 (A28) and a protein of the thaumatin family (A34). Tags A18, A22 and A35 matched with the same set of lentil and other legumes gene sequences and identified the same gene functions, CORA G7K7D2_MEDTR and the glycine-rich protein Q0E7L3_PEA. Tag A22 corresponds to a sense sequence, conversely, tags A18 and A35 are identical to antisense sequences, suggesting a possible simultaneous transcription from opposite promoters in this gene which could generate natsiRNAs.

A set of 52 tags were exclusive or overrepresented in the tolerant RILs; of which 29 identified lentil sequences (named as T). A set of the identified functions from these sequences (Additional files 9 and 17) is included in the general responses to cold and drought abiotic stresses (dormancy-associated proteins, dehydrins and CORA proteins), including a sequence (T17) similar to a cold-acclimation specific protein in barrel medic (B1NY79_MEDTR) and pea (O64396_PEA), or to other abiotic stresses, e.g., the stress responsive A/B barrel domain protein (T7). As in set A of tags, some T tags identified sense and antisense transcripts related to CORA proteins. Other sequences were similar to transcription factors, Myb or a zinc finger CONSTANS-like (A0A072VQB9_MEDTR) (at least some zinc finger family of transcription factors are related to abiotic stress response mediated by abscisic acid [28]).

When the susceptible RILs were considered a total of 61 tags were exclusively observed or overrepresented. Forty of them (named as S) identified lentil nucleotide sequences (Additional files 10 and 18). This tag set shares similarity with the set from the non-acclimation set (N) since many of the identified sequences are from the chloroplast genome and/or related to chloroplastic functions. In fact both sets (S and N) shared many tags that identified the same sequences (See end of Additional file 13), thus the chloroplastic functions would be particularly representative of non-acclimated frost-susceptible lentil plants. Other tag sets identified gene sequences related to cell wall components (e.g., transmembrane proteins) and their biosynthesis (e.g., pectinacetylesterase, caffeic acid 3-O-methyltransferase). Another noticeable group is constituted by sequences that are likely involved in transcription control and signal transduction (MYB-like factor, Zinc finger CCCH, Protein phosphatase 2C). Lastly, tag (S21 = A28) identified the resistance gene *PR4* of lentil; this gene has been related to the defense against *Ascochyta lentis* [29].

The joint analysis of these last four sets of tags revealed that several tags are common to two sets. This fact was particularly evident among the non-acclimated (N) and the susceptible (S) sets, and the acclimated (A) and tolerant (T) sets. Thus these tags were added to the NAS and AT sets, respectively. The first group is mainly related to photosystem functioning while the second was mainly related to genes coding for glycine-rich proteins.

Likewise, the joint analysis of data suggests that chilling response is related to differential expression of proline-rich, transmembrane, cold-acclimation specific protein and LEA proteins, thaumatins and pathogenesis related proteins. Proline-rich sequences and the transmembrane sequences would be overexpressed in the acclimated lentil plantlets, and more in the tolerant RILs (AT) than in susceptible ones (AS) within the acclimated. In the tolerant RILs, different subsets of the transmembrane proteins seem to be overexpressed in the acclimated and non-acclimated plants (Table 4).

**Table 4:** Tags related to proline-rich (PR) and transmembrane (TM) sequences and their distribution

	NAS	NAT	AS	AT	Type
CATGATGTTTTTAAGTAAATATGTAA A2 = T23		0.25	4.67	**106.34** ^a^	PR
CATGGGATATATATTCTTGTAATCTT A5	0.47	1.50	59.73	**365.82**	PR
CATGCCTTTTGATTTTATTATCAGCA^b^ A6	0.24	4.49	54.31	**679.82**	PR
CATGTTGTCCATTGATAAAAGGACTC^c^ A10		0.25	2.24	**16.72**	PR
CATGTTCCCAAACCACCTGTTGCCCC A14			0.56	**30.76**	PR
CATGAATATAAAAAATACTTGAGATG AT1				**1102.15**	PR&TM
CATGAATATAAGAAATACTTGAGATG S25	45.97	13.46	**313.57**		PR&TM
CATGTAATAGTATTGTGATTTCTGCT A21 = S6	0.24	0.25	**16.99**		TM
CATGTTATGAATGAAAAATTACGTGT AT9				**59.19**	TM
CATGAAGCATTTTTAGAATGTAAAAA AT18				**30.76**	TM
CATGTTTATTATTTTTATAAAATTAT A17 = T15		0.50		**24.74**	TM
CATGCAATAAATCTATAAATTATATC NAT33		**20.19**			TM
CATGGAAGAATGTTTACTTGTTCTTC NAT42		**15.95**			TM
CATGAAGAAAAATGAGACAAGATCTT S9	**24.40**		6.16		TM

^a^Numbers in bold indicate the highest value for each tag, normalized values. Empty cells indicate zero observations

^b^A6 and A10 identified the same lentil sequence in two points along it sequence

^c^See also the transmembrane sequences referred in Table 2

Chilling response increases the expression of some cold-acclimation specific protein and LEA proteins (Table 5). Tag AS23 was exclusively observed in the acclimated susceptible lentil RILs, but with a relatively low normalized value (35.84), while tag T17 was observed in all sets but was predominant in the tolerant RILs (193.67 in NAT and 1154.99 in AT versus 0.24 in NAS and 29.49 in AS). Tags AS29 and A24 identified sequences with homology to LEA proteins, such as LEA18 of *A. thaliana*. The responses seem to be genotype dependent (e.g., AS29 and AT2), and the tolerant genotypes express at least some of these proteins at higher rates than susceptible genotypes.

**Table 5:** Expression levels of cold-acclimation specific^1^ and late embryogenesis abundant^2^ proteins

Tag name	Tag sequence	NAS	NAT	AS	AT
AS23^1^	CATGGCAATAGCAGCAGCAGTGATAG			35.84^a^	
T17^1^	CATGATAGCAGCAGCAGTGATAGTGA	0.24	193.67	29.49	1154.99
A30^1^	CATGACTTGTATTTGTCTAAGACTGA	4.03	4.98	37.33	198.29
AS29^2^	CATGTTTATGTGATCCATTCACTAAA			24.26	
AT2^2^	CATGGACGCAATGCTCATTACCAGAA				138.77
A24^2^	CATGGAGCTTTTAGACAAAGCCAAAA	0.47	2.99	18.66	95.97
S39^2^	CATGTGGAATTTAAAAGTTAGGCTTA	3.32	2.49	38.64	

^a^Normalized values. Empty cells indicate zero observations

Tags related to pathogenesis related proteins appeared in different tag sets. The two different lentil PR5-like sequences appeared only in the non-acclimated tolerant set (NAT); another thaumatin-like protein seemed to be over expressed in the acclimated tolerant RILs (Table 6). On the contrary, the other PR sequence identified, PR4, was over expressed in the acclimated susceptible RILs. Thus while expression of PR5 and the other thaumatin-like sequences seem to be characteristic of the tolerant RILs, the PR4 sequence seems to be characteristic of the susceptible ones.

**Table 6:** Tags related to thaumatin and PR sequences

	NAS	NAT	AS	AT
CATGTGTTTTCAGTTAATGAATTAAT NAT16, Thaumatin, PR5		41.38^a^		
CATGCCCAGGTGGTACCAATTATAGA NAT22, Thaumatin-like protein PR-5a		32.15		
CATGAAGTTTTTTTAATAAAACTTCA A34, PR Thaumatin-like	2.13		3.36	31.77
CATGTATACTTACTTAATTGAAAGAA A28 = S21, PR4		1.49	46.10	

^a^Normalized values. Empty cells indicate zero observations

## Discussion

According to the experimental procedure used, different genotypes should be represented in each RNA bulk (susceptible and tolerant) since different RILs are pooled in each one. But the rationale underlying this procedure is that tolerant and susceptible parents contribute positive and negative QTLs to the trait and the corresponding genes are characterized by their expression profile and allelic state, and the same beneficial and detrimental gene alternatives, respectively, accumulate in the extreme best and worse individuals of a segregating population. Pooling of extreme individuals reunites beneficial genes (alleles) in the best-performing bulk, and detrimental genes (alleles) in the worst-performing one, while, on average, they are similar for the non-relevant genes. The transgressive segregation for frost response showed by the lentil RILs (Fig. 2) agrees with the expected segregation for a quantitative character under the control of several quantitative genes [11]. The same should be true for the expression profile, pooling of extreme individual would highlight differences in gene expression related to the response.

A prove that tag normalized numbers are useful to estimate gene expression levels is afforded by tags from the same transcript. As was above mentioned, the Super-SAGE technique can generate two or more different tags along a single RNA sequence, but tags are usually more frequents as they are closer to the 3′ transcript end. But even so, if normalized numbers are related to the expression level the increase rate can be similar within the tags from a same sequence in different treatments. For instance, tags A6 and A10 correspond to the same sequence, A6 is in the 3’UTR while A10 is in the ORF; A10 is less frequent than A6; but the ratios between the treatments for each tag are similar (Additional file 8).

A large proportion (~ 48%) of the functions putatively over expressed in the susceptible RILs growing at normal conditions (NAS) are probably functions related with plantlets growing at fast rate under good conditions of temperature and light (Additional file 13). They were functions related to photosynthesis and chloroplast metabolism, to general metabolism, to signal transmission, to protein folding, modification and transport, and coding for histones. Some of the functions could be related to stress response but at least some of them are also related to other functioning, for instance, GDSL esterase/lipases are also related to plant growth and development [30, 31]. Likewise, ascorbate peroxidases are related to stress responses, but they are also involved in normal growth and plant development [32]. Lastly, quinone oxidoreductases are part of the metabolic machinery protecting against oxidative stress. We have found three tags (NAS22, S32 and CATGAAAACCATTCAAAAGATGTACG, found in a further search) which identified three different polypeptides of the NADH-quinone oxidoreductase complex. The most relevant fact was that none of these tags was found in the acclimated tolerant bulk (AT). This result suggests that down regulation of these oxidoreductases would help in the response to frost temperatures.

Comparing these previous results with those from tolerant RILs growing at the same normal conditions (NAT) some function represented among the putatively over expressed genes are common: photosynthesis and chloroplastic and mitochondrial functions, although less numerous, protein synthesis and posttranslational metabolism, general metabolism and signal transduction. However, in the NAT set of results the putative functions related to stress response are more numerous and more clearly related to these responses. Some of them can be also related to other functions involved in growth and general metabolism, but other are more directly related to stress response, such as pathogen related (PR) genes (at least some PR genes have proved to be also related with responses to abiotic stresses [33]), a cold and drought-regulated protein gene and a gene related to vernalization, or aluminum (the sequence related to an aluminum-induced protein observed in this bulk (NAT2 = NAT17) is different from the one observed in the AT bulk (AT23)). These results raise the question whether these genes are also implicated in normal growth and metabolism or if they are over expressed in frost tolerant genotypes even at not stressing temperatures so that these plants are better preadapted to stressing conditions.

Like in the NAS set, the AS tag set identified several sequences related to photosynthesis and other chloroplast functions, to general metabolism, to signal transmission, to protein folding, modification and transport, and to cell wall and membrane components and transport. Tag AS9 was identified as one of these membrane components, an aquaporin sequence. Although the expression of aquaporins has been repeatedly related to drought and cold responses in plants, they are also associated to plant growth [34], and cold treatment results in a complicated transcriptional regulation pattern for various aquaporins, at least in some plant species [35]. Thus, the association of this particular lentil protein to chilling response is not granted. ABC proteins are membrane-intrinsic primary active pumps. In higher plants, ABC proteins constitute a large family, grouped into eight subfamilies ABCA–ABCI (except ABCH) [36]. Some ABCGs contribute to the intercellular transport of the stress hormone ABA and therefore they are related to stress. The called multidrug resistance or pleiotropic drug resistance proteins (see NAT 12 and NAT 19) are member of the ABCG subfamily [36].

Tags AS23 and AS29 were related to cold-acclimation specific and late embryogenesis abundant proteins, respectively (Table 5). Late embryogenesis abundant (LEA) proteins are associated with dehydration tolerance and freezing response in many plant species, and there is compelling evidence for a functional role of at least some LEA proteins in cellular stress tolerance. The expression of the cold-acclimation specific protein 15 from *M. sativa* and the Peaci11.8 from pea are correlated with cold response [37]. Two tags (AS23 and T17) identified lentil sequences with homology to these two genes and others for cold-acclimation specific proteins in other legume species. The two lentil tags correspond with two different sequences, although all except one lentil sequence (gi 331109117) in databases were incomplete. The analysis of aligned sequences from lentil and other legume species indicate that there are several sequences per species, and a relative high degree of variability among sequences. A tag (AS29) exclusively found in the AS set identified a sequence with homology to LEA18. In *A. thaliana* LEA18, as other LEA proteins, are related to membrane stability [38]. Another lentil LEA sequence tag (A24) with homology to LEA14 was over represented in both acclimated susceptible and tolerant RILs (Table 5). Among the identified sequences in the AT set of tags several tags were related to chloroplastic functions and many others were related or potentially related to stress response. Tag AT2 (Table 5) identified a putative ABA-induced guard cell protein that also shares homology with LEA proteins from *Medicago* (G7ISC1_MEDTR). AT17 was related to an acyl-carrier protein; a peanut acyl carrier protein has been related to cold response, and its ectopic expression in tobacco alters resistance to cold stress in addition to the fatty acid composition in the leaf [39].

Another function related to stress responses is phospholipase D. Phospholipases D (PLDs) are major lipid degrading enzymes that hydrolyze phospholipids to produce phosphatidic acid. They affect not only cellular membrane structure and stability, but also regulate many cellular functions. PLDs are important components in the sensory system that decodes stress-related signals in association with microtubules [40]. Plant microtubules function in the perception of mechanical membrane stress and its derivatives, such as osmotic or cold stress and thus related to the cold stress response [40]. Increasing evidence shows that PLDs play pivotal roles in signaling plant responses to various stress cues, such as water deficit, freezing and salinity, as well as the stress hormone abscisic acid (ABA). In *Arabidopsis thaliana* genetic knockout of the plasma membrane–associated PLDδ renders plants more sensitive to freezing, whereas overexpression of PLDδ increased freezing tolerance. The PLDδ alterations did not affect the expression of the cold-regulated genes COR47 or COR78 or alter cold-induced increases in proline or soluble sugars, suggesting that the PLD pathway is a unique determinant of the response to freezing [40–42]. Two different phospholipases D sequences were identified in lentil, one among the acclimated tolerant RILs (AT26) and the other among the acclimated susceptible (AS11). Both were putatively overexpressed in response to chilling, but it seems that the gene overexpressed would be genotype dependent. Furthermore, in our data we have found a putative pair of alleles of an actin-depolymerizing protein (cofilin) which would be exclusive of the tolerant (CATGTCTTGGATACTAAAATCCATTG) or the susceptible (CATGTCTTGGATACTAAAATCCATCG) RILs, respectively. This suggests that these particular alleles could be related to the differential adaptation to frost temperatures or to be tightly linked to an important gene related to that response. In an additional search we found a tag which identified a different lentil sequence with homology to an actin-depolymerizing factor (G7ZZK8_MEDTR). The normalized values of this tag were relatively high (between 50.35 and 11.34) except in the acclimated susceptible bulk in which it was absent. This fact support that the expression of these genes coding for actin-related proteins could be related to the cold response in lentil. At least in rice, there are evidences that the actin-depolymerizing gene family participate in plant abiotic stresses response [43].

S-adenosylmethionine decarboxylase proenzyme is involved in the synthesis of polyamines, and polyamines have been related to the response of abiotic stress such as drought and cold in plants. At the transcriptional level in *Arabidopsis*, the expression of this enzyme gene is induced by different abiotic stresses, and its overexpression often correlates with enhanced tolerance to different types of stress [44]. It seems that genes of this family are over-expressed in the lentil tolerant acclimated bulk when comparing to the susceptible acclimated bulk. In addition to the S-adenosylmethionine decarboxylase proenzyme identified by tag AT12 (exclusively observed in the acclimated tolerant bulk), a second enzyme of this type was identified in an further search; the corresponding tag (ATGTACTAGTGTCTTAGTAATTTTA) showed normalized values of 27.25 in the NAS bulk, 162.01 in the NAT one, 369.50 in the AT, being absent in the AS.

Several observable differences among the four lentil tag sets are related to the distribution of the tags identifying sequences for glycine-rich proteins (GRPs), also designated as cold and drought-regulated protein (CORA) or dormancy-associated protein; and also for proline-rich proteins (PRPs). In plants, glycine-rich proteins GRPs are characterized by the presence of semi-repetitive glycine-rich motifs. In general, their expression patterns are regulated and the expression is modulated by biotic and abiotic factors. Plant GRPs are classified based on their general structure, taking into consideration the arrangement of the glycine repeats as well as the presence of conserved motifs such as a RNA-recognition motif (RRM) or a cold-shock domain (CSD), among others [45]. Thus, at least GRPs with RRM (GR-RBPs) can be involved in RNA metabolism in stress response of plants [46]. Cyanobacterial, plant and metazoan GR-RBPs have been implicated in the responses to changing environmental conditions, particularly to cold stress. Expression analysis of plant GR-RBPs has revealed that at least some of the genes are strongly up regulated by cold stress [46, 47]. In our experiment tags identifying these gene sequences were observed in all four tag sets but they were predominant in the acclimated tolerant (AT) lentil bulk and some of them in the non-acclimated tolerant (NAT) (Table 2). Thus, it seems that the level of overexpression of the corresponding genes would be a characteristic of the tolerant genotypes, so that tolerant genotypes would over express some of the GRPs even at “optimal” conditions (non-acclimated) and more of them in response to chilling. This over expression would contribute to a preadaptation of the tolerant genotypes to frost temperatures increasing their survival.

Proline-rich proteins (PRPs) are cell wall and plasma membrane-anchored factors involved in cell wall maintenance and its stress-induced response, some of them have transmembrane domains. Most evidences of differential expression of this heterogeneous group of proteins are in relation to salt or drought stresses [48]. Some of these proline-rich and transmembrane proteins seem to be differentially expressed in lentil in response to chilling (Table 4). The general trend of these proline-rich sequences and the transmembrane sequences would be overexpression in the acclimated lentil plantlets, and more in the tolerant RILs (AT) than in susceptible ones (AS) within the acclimated. In the tolerant RILs, different subsets of the transmembrane proteins seem to be overexpressed in the acclimated and non-acclimated plants. Thus, frost tolerance in lentil, like in other plant species, seems be associated to the differential expression of characteristics sets of glycine-rich protein, proline-rich protein and transmembrane protein genes [6, 45, 46].

Secreted pathogenesis-related (PR) proteins with antifreeze activity such as thaumatin-like proteins have been isolated from several plant species [33]. It is known that this kind of proteins is expressed in response to both biotic and abiotic stresses and play other roles in plant. For instance, PR4A protein from *Lens culinaris* is involved in the defense against *Ascohyta lentis* [29]. A thaumatin-like protein, Rj4, that belongs to pathogenesis-related (PR) protein family 5 controls nodule symbiotic specificity in soybean [49]. On the other hand, defense-related proteins revealing similarity to thaumatin increased in relative abundance upon chilling treatment in both frost-tolerant winter wheat and frost-sensitive spring wheat [50].

There are apparently puzzling results in relation to the high number of stress related functions (glutathione peroxidase, aluminum-induced protein, thaumatin-like protein, Cu-Zn superoxide dismutase, etc.) detected in the tolerant RILs under non stressing conditions (non-acclimated, NAT). These results point to the complicated relationships between stresses and the expression of different functions related to stress response. Thus, the level of glutathione peroxidase (Gpx) mRNA and activity from various organisms is affected by stress conditions. In several studies, the Gpx expression or activity is generally up-regulated in response to stress. However, there are exceptions to this pattern [51]. Glutathione transferases belong to a superfamily of multifunctional enzymes known for their ability to bind and sequester an array of hydrophobic compounds of endogenous and xenobiotic origin [52]. The expression of some glutathione transferases has demonstrated to be correlated to cold sensitivity in two rice subspecies and in tobacco transformants [52, 53]. A type II metallothionein gene, *CmMet-*2 (GQ900702), from *Cicer microphyllum*, a high altitude cold desert-adapted wild relative of cultivated chickpea, is expressed in roots and aerial plant parts; transcripts were induced in all parts of plants in response to cold stress at 4 °C; and the transcript abundance was found to increase exponentially with time course from 6 to 24 h after exposure. Other tests also suggest the involvement of *CmMet-2* in multiple stress response [54]. A metallothionein-like protein was exclusively identified in the lentil NAT set; however other metallothioneins are highly and similarly expressed in the four lentil tag sets (NAS, NAT, AS, AT). The results in this experiment with lentil raise again the question on whether lentil cold tolerant genotypes express stress-protectant functions even in absence of stress, thus pre-adapting them to stress conditions and improving their survival rate. In *Arabidopsis* the down-regulation of copper/zinc superoxide dismutases (related to oxidative stress) and a copper chaperone for these enzymes increase heat tolerance [55]. In lentil, two tags identifying a copper/zinc superoxide dismutase (NAT32) and a superoxide dismutase copper chaperone (NAT43) were only observed in the non-acclimated tolerant bulk, but were not observed after acclimation, pointing to that probably the down-regulation of these proteins would also help in the response to cold.

The mechanisms involved in cold acclimation included, among others, modifications of plant cell membranes, changes in photosynthesis levels and photosynthesis-related component, changes in reactive oxygen species levels and non-enzymatic antioxidants, and differential expression of several gene sets such as cold-responsive genes. Chloroplast is the first and most severely affected organelle when plants are exposed to abiotic stresses such as freezing exposure and it is the target of many cold acclimation processes which are the results of the chloroplast-nucleus cross-talk [6–9]. In pea freezing tolerance seems to rely on a higher inherent photosynthetic potential at the beginning of the cold exposure, combined with an early ability to start metabolic processes aimed at maintaining the photosynthetic capacity [8]. In our results, it is clear that each bulk set is characterized by a putative over expression of sets of genes (either from the nuclear genome or the chloroplast genome) related to photosystem, CO_2_ fixation and in general chloroplast functions; in many instances it seems that some gene products probably substitute other similar products from the same gene family. This fact suggests that some gene family variants are better in some environmental condition than other variants. In particular transcripts from the chloroplast genome were abundant among the susceptible RIL genotypes. Another important component in cold acclimation is transmembrane proteins, also related to the proline-rich proteins. Results in Table 4 point to the fact that, in general, these proteins would be over expressed in the tolerant acclimated bulk (AT). A similar over expression of glycine-rich proteins would be also characteristic of the AT bulk (Table 2). Among the cold-responsive genes different members of a gene-family of a zinc finger CONSTANS-like sequence were observed in the tolerant or susceptible RILs. CONSTANS-like proteins (included in the B-box protein family, a class of zinc-finger transcription factors) are key factors in regulatory networks controlling growth and responses to biotic and abiotic stresses [28, 56]. Furthermore, cold-acclimation specific and late embryogenesis abundant proteins seem also to be generally overexpressed in the acclimated tolerant bulk.

## Conclusions

This set of results in the differential expression in lentil in relation to cold acclimation points to that, at least the expression of genes for proline-rich, glycine-rich, and transmembrane proteins as well as genes related to photosynthetic functioning could be important in the response to frost temperatures, contributing to a differential lentil survival against challenging frost temperatures. To sum up, we have provided a set of candidate genes implicated in the response to frost in lentil, which represents useful information for deeper and more detailed researches on this subject.

## Additional files

Additional file 1: Differential response of the two lentil parent genotypes to the treatment at −9 °C. The tolerant WA8649041 is on the left and the susceptible Precoz on the right. (JPEG 205 kb)

Additional file 2: Differential response of some RILs to a treatment at −15 °C after 15 days of recovering. Note that while in most of the RILs all plantlets died, in one RIL the plantlets survived with little damage, and in some RILs a few plants survived but showing a lower growth. (JPEG 214 kb)

Additional file 3. Lists of significant Super-SAGE tags. Exclusive significant SuperSAGE tags (fold change >4) observed in acclimated lentils of the frost-susceptible RILs (AS). (XLSX 16 kb)

Additional file 4: Lists of significant Super-SAGE tags. Exclusive significant SuperSAGE tags (fold change >4) observed in acclimated lentils of the frost-tolerant RILs (AT). (XLSX 13 kb)

Additional file 5: Lists of significant Super-SAGE tags. Exclusive significant SuperSAGE tags (fold change >4) observed in non-acclimated lentils of the frost-susceptible RILs (NAS). (XLSX 15 kb)

Additional file 6: Lists of significant Super-SAGE tags. Exclusive significant SuperSAGE tags (fold change >4) observed in non-acclimated lentils of the frost-tolerant RILs (NAT). (XLSX 14 kb)

Additional file 7: Lists of significant Super-SAGE tags. Significant over represented SuperSAGE tags (fold change >4) from non-acclimated lentil plants considering both genotypes – susceptible and tolerant – together (N). (XLSX 13 kb)

Additional file 8: Lists of significant Super-SAGE tags. Significant over represented SuperSAGE tags (fold change >4) from acclimated lentil plants considering both genotypes – susceptible and tolerant – together (A). (XLSX 15 kb)

Additional file 9: Lists of significant Super-SAGE tags. Significant over represented SuperSAGE tags (fold change >4) from the tolerant lentil RILs considering both treatments together (T). (XLSX 15 kb)

Additional file 10: Lists of significant Super-SAGE tags. Significant over represented SuperSAGE tags (fold change >4) from the susceptible lentil RILs considering both treatments together (S). (XLSX 16 kb)

Additional file 11: List of the identified functions related to the differentially expressed tags. SuperSAGE tags exclusively observed in acclimated lentils of the frost-susceptible genotypes (AS) and the corresponding sequences and gene functions identified. (XLSX 18 kb)

Additional file 12: List of the identified functions related to the differentially expressed tags. SuperSAGE tags exclusively observed in acclimated lentils of the frost-tolerant genotypes (AT) and the corresponding sequences and gene functions identified. (XLSX 16 kb)

Additional file 13: List of the identified functions related to the differentially expressed tags. SuperSAGE tags exclusively observed in non-acclimated lentils of the frost-susceptible genotypes (NAS) and the corresponding sequences and gene functions identified. (XLSX 19 kb)

Additional file 14: List of the identified functions related to the differentially expressed tags. SuperSAGE tags exclusively observed in non- acclimated lentils of the frost-tolerant genotypes (NAT) and the corresponding sequences and gene functions identified. (XLSX 19 kb)

Additional file 15: List of the identified functions related to the differentially expressed tags. SuperSAGE tags from non-acclimated lentil plants considering both genotypes – susceptible and tolerant – together. (XLSX 14 kb)

Additional file 16: List of the identified functions related to the differentially expressed tags. SuperSAGE tags from acclimated lentil plants considering both genotypes – susceptible and tolerant – together. (XLSX 15 kb)

Additional file 17: List of the identified functions related to the differentially expressed tags. SuperSAGE tags from the tolerant lentil genotypes considering both treatments – acclimation and control – together (XLSX 15 kb)

Additional file 18: List of the identified functions related to the differentially expressed tags. SuperSAGE tags from the susceptible lentil genotypes considering both treatments – acclimation and control – together. (XLSX 16 kb)

Additional file 19: Additional list of functions. Summary of representative groups of related functions identified by the different set of lentil tags. (XLSX 13 kb)

## Abbreviations

A: acclimated
AS: acclimated susceptible
AT: acclimated tolerant
BLAST: Basic Local Alignment Search Tool
CBF: C-repeat binding factors
COR: cold-responsive genes
CORA: cold and drought-regulated protein
FT: freezing tolerance
LEA: late embryogenesis abundant protein
N: non-acclimated
NAS: non-acclimated susceptible
NAT: non-acclimated tolerant
PR: pathogenesis related
PRP: Proline-rich proteins
QTL: quantitative trait loci
RIL: recombinant inbred line
S: susceptible
SAGE: Serial Analysis of Gene Expression
T: tolerant.
