# Supplementary figures and images for: Deep Super-SAGE transcriptomic analysis of cold acclimation in lentil (Lens culinaris Medik.)
Source: BMC Plant Biol. 2017 Jun 30;17:111. doi: 10.1186/s12870-017-1057-8 (PMC5493078; doi:10.1186/s12870-017-1057-8)

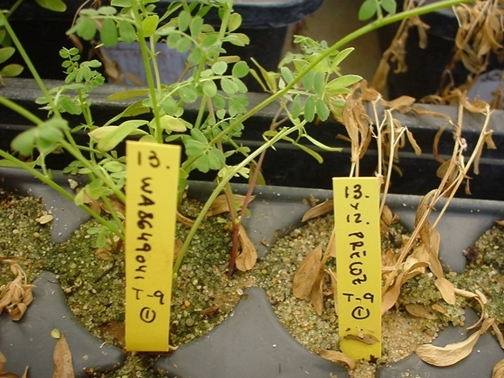

Supplement: Supplementary file 1 — Differential response of the two lentil parent genotypes to the treatment at −9 °C. The tolerant WA8649041 is on the left and the susceptible Precoz on the right. (JPEG 205 kb) [file 12870_2017_1057_MOESM1_ESM.jpg]

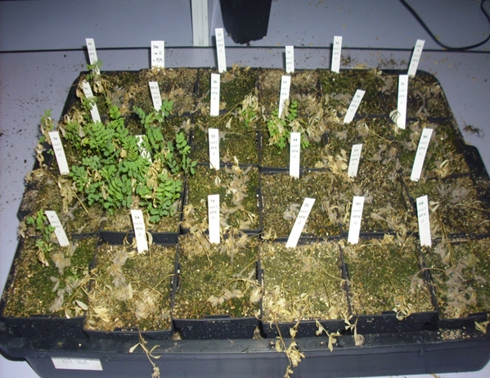

Supplement: Supplementary file 2 — Differential response of some RILs to a treatment at −15 °C after 15 days of recovering. Note that while in most of the RILs all plantlets died, in one RIL the plantlets survived with little damage, and in some RILs a few plants survived but showing a lower growth. (JPEG 214 kb) [file 12870_2017_1057_MOESM2_ESM.jpg]
